# Reversibility of the Enlargement of the Pulmonary Artery in COVID-19 Pneumonia as a Marker of Remission of the Disease

**DOI:** 10.3390/jpm14020161

**Published:** 2024-01-31

**Authors:** Andreas M. Matthaiou, Nikoleta Bizymi, Konstantinos Pagonidis, Eirini Manousaki, Michail Fragkoulakis, Irini Lambiri, Ioanna Mitrouska, Eirini Vasarmidi, Nikolaos Tzanakis, Katerina M. Antoniou

**Affiliations:** 1 Laboratory of Molecular and Cellular Pneumonology, Medical School, University of Crete, 71500 Heraklion, Greece; nikoletabizymi@yahoo.gr (N.B.); eirvasar@gmail.com (E.V.);; 2 Department of Respiratory Medicine, University Hospital of Heraklion, 71500 Heraklion, Greece; 3 Department of Medical Imaging, Knossos Medical Diagnosis Centre, 71409 Heraklion, Greece; kpagonidis@yahoo.de (K.P.);

**Keywords:** COVID-19, pulmonary artery enlargement, pulmonary hypertension, chest imaging

## Abstract

Coronavirus disease 2019 (COVID-19) pneumonia is associated with extensive pulmonary microangiopathy and the enlargement of the pulmonary artery (PA), while its progression after the remission of the disease has not been investigated yet. The aim was to assess the diametral increase in the PA in COVID-19 pneumonia, as revealed on chest computed tomography (CT), and further investigate its progression. This was a retrospective cohort study of patients with COVID-19 pneumonia, without prior history of pulmonary hypertension, who underwent CT pulmonary angiography before, during, and after the infection. Pulmonary embolism was excluded in all cases. The main PA diameter (MPAD) was assessed in consecutive chest imaging. Statistical analysis was performed with the non-parametric Wilcoxon and Kruskal–Wallis tests, while correlations were performed with the non-parametric Spearman test. A mean ± SD MPAD of 3.1 ± 0.3 cm in COVID-19 pneumonia was significantly decreased to 2.8 ± 0.3 cm in the post-infectious state after 2–18 months in 31 patients (*p*-value: <0.0001). In a subgroup of six patients with more than one post-COVID-19 CT, a significant further decline in the diameter was observed (*p*-value: 0.0313). On the other hand, in accordance with the literature, a significant increase in the MPAD during COVID-19 pneumonia was noted in a group of 10 patients with a pre-COVID-19 CT (*p*-value: 0.0371). The enlargement of the PA is a common finding in COVID-19 pneumonia that regresses after the remission of the disease, indicating that this reversible cardiovascular event is a potential marker of disease activity, while its course in long COVID is yet to be determined.

## 1. Introduction

Coronaviruses have been historically described as one of the most common causes of viral respiratory tract infections. In 2002 and 2012, two novel coronavirus species of zoonotic origin, i.e., severe acute respiratory syndrome-related coronavirus (SARS-CoV-1) and Middle East respiratory syndrome-related coronavirus (MERS-CoV), respectively, were responsible for large-scale outbreaks of viral pneumonia and acute respiratory distress syndrome (ARDS) with high mortality. A third species, SARS-CoV-2, appeared at the end of 2019 in China, rapidly spread around the world, and caused the coronavirus disease 2019 (COVID-19) pandemic. Most patients present with fever, cough, and dyspnoea, as well as headache, myalgia, and diarrhoea, and may develop viral pneumonia with a severe clinical course, including respiratory failure, ARDS, multiorgan failure, and even death. A central stage in the pathogenesis of this disease is the virus-induced cytokine storm and the consequent vascular endothelial dysfunction. Thromboembolic events, including pulmonary embolism, are some of the commonest complications [1,2].

The increasing amount of scientific evidence about the deleterious effects of SARS-CoV-2 on pulmonary and systemic vasculature, the multiple well-described mechanisms by which the virus appears to cause vascular dysfunction, and the clear association of the infection with both micro- and macroangiopathy have reasonably led many experts to characterise COVID-19 as a vascular disease. The pulmonary vasculature, in particular, demonstrates outstanding derangements during the infection, including extensive microvascular thrombosis, microangiopathy, angiogenesis, and endothelial activation. Both direct viral insult and systemic inflammatory response lead to pulmonary endothelial cell dysfunction. The resultant disturbance of endothelial homeostasis, accompanied by the activation of the coagulation cascade and inhibition of fibrinolytic pathways, are responsible for the diffuse microvascular and macrovascular thrombotic events in COVID-19 that are especially evident in the pulmonary circulation [3,4]. Pulmonary artery (PA) thrombosis is a potential complication in severe COVID-19 despite the administration of anticoagulation treatment [5]. In accordance with the theory that endothelium damage is caused by SARS-CoV-2 are several post-mortem autopsy studies, in which diffuse alveolar damage and atypical cells that are thought to be endothelial cells infected via the human angiotensin-converting enzyme 2 receptor (hACE2R) are common findings [6,7,8,9].

Cardiovascular complications are some of the most common sequelae of COVID-19 and significantly increase the risk of mortality for its patients. Myocardial injury is thought to be a result of systemic inflammation, as well as the direct attack of the myocardial cells by the virus [10]. Pulmonary small vessel vasoconstriction, attributed to both elevated prostaglandin levels and hypoxemia, leads to an increase in the pulmonary arterial pressure and subsequent right cardiac chamber dilatation [11,12].

In critically ill COVID-19 patients, pulmonary and systemic inflammation lead to an increase in the right ventricular (RV) afterload and subsequent myocardial injury, reflected by the elevation of serum myocardial enzyme levels and the demonstration of RV strain imaging signs. Although cardiac magnetic resonance imaging is the gold standard method for the evaluation of RV function, bedside echocardiography remains the most commonly used diagnostic tool for the determination of the RV strain, especially during the COVID-19 pandemic. While conventional RV function parameters, i.e., RV fractional area change (RVFAC) and tricuspid annular plane systolic excursion (TAPSE), are associated with higher mortality in COVID-19, the RV longitudinal strain (RVLS), as determined by means of two-dimensional speckle-tracking echocardiography (2D-STE), has been found to predict a higher risk of mortality more accurately than the other echocardiographic indices [13]. Li et al. and Gao et al. used bedside echocardiography in their cohorts of patients and noted that, according to clinical and biochemical data, patients with more severe disease tended to have more enlarged cardiac chambers and PA and a higher pulmonary artery systolic pressure (PASP) [12,13].

Well established is the importance of medical imaging for the diagnosis, estimation of disease severity, and evaluation of disease progression in COVID-19. Chest computed tomography (CT) can reveal no abnormalities in mild cases and ground-glass opacities (GGOs) in most cases, whereas fibrotic-like and fibrotic changes can be demonstrated on follow-up chest imaging in more severe cases [14,15]. Interestingly, intrapulmonary vascular enlargement in areas of GGOs in COVID-19 pneumonia has been described as an ancillary chest CT finding in about 42% of patients and associated with worse clinical features on admission. Notably, this finding was observed in both dependent and non-dependent areas of lung parenchyma, differentiating it from the dependent vascular redistribution that is typically seen in heart failure [16]. Furthermore, besides delineating in detail the extent of lung parenchymal involvement, chest CT can also reveal abnormal findings regarding the pulmonary vasculature and cardiac chambers in COVID-19.

In 2020, Spagnolo et al. were the first to observe and report an acute increase in the PA diameter, as demonstrated on chest CT of patients with COVID-19. They were then followed by several other research groups further describing and establishing the association of the disease with an enlargement of the PA, as well as correlating this finding with clinical features and prognostic parameters [17]. However, to the best of our knowledge, no data of PA metrics during post-COVID-19 follow-up chest imaging were reported in those studies. We hypothesised that the enlargement of the PA, which is only acutely observed in COVID-19, could regress after the remission of the disease, as the driving forces of systemic inflammation and hypoxemia, which contribute to increased pulmonary vascular resistance and a resultant increase in right heart afterload and the dilatation of right cardiac chambers, subside after the resolution of COVID-19 pneumonia.

## 2. Materials and Methods

**Figure 1:**
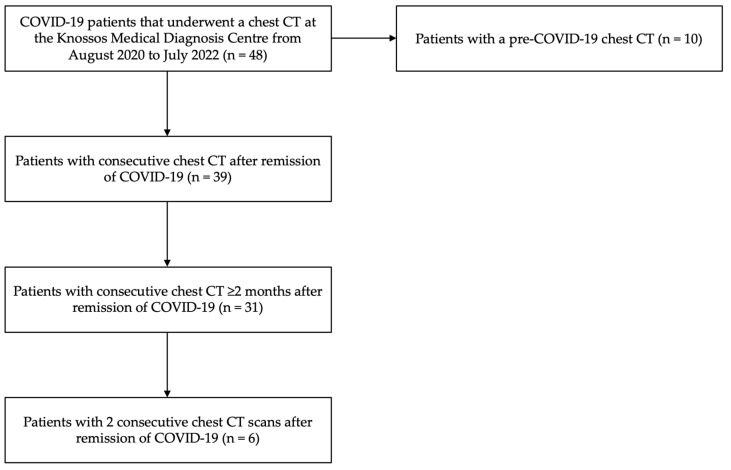
Flow diagram of the participants of the study. The diagram shows the process of selecting the individuals enrolled in this study.

This was a retrospective cohort study investigating the structural alterations in the PA on chest imaging in patients with COVID-19 pneumonia. The study participants were diagnosed with COVID-19 by means of molecular testing, i.e., reverse-transcriptase polymerase chain reaction (RT-PCR) for SARS-CoV-2, in the period from August 2020 to March 2022, and were selected from a pool of 48 patients that underwent a chest CT in the acute phase of the infection. Individuals with previously known pulmonary hypertension (PH) were excluded. In 39 patients from the total pool, consecutive chest imaging was implemented after the remission of the disease, while in 31 of them, the repeat scan was performed at least 2 months after the acute phase of the infection. In 6 patients of this study group, at least two serial post-COVID-19 scans were performed to consecutively assess the course of abnormal imaging findings. Additionally, in 10 patients from the total pool, a pre-COVID-19 scan was also available. Τhe groups of the enrolled individuals are schematically given in the flow diagram below (Figure 1).

Medical imaging examinations were conducted at the Knossos Medical Diagnosis Centre in Heraklion, Crete, Greece, from August 2020 to July 2022, based on clinical indications used most commonly for the investigation and reassessment of lung parenchymal changes and pulmonary vascular defects, especially pulmonary embolism. Since pulmonary artery thrombosis can appear even in patients receiving anticoagulation treatment, its exclusion in our study group was crucial. All participants provided their written consent for their inclusion in the study, which followed the principles of the Declaration of Helsinki.

**Figure 2:**
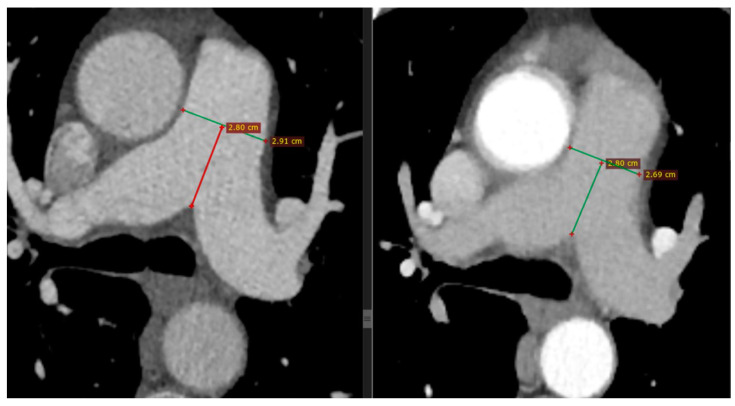
Representative chest CT images. Consecutive measurements of MPAD at mid-PA at the level of the mid-aortic arch and main carina on chest CT in a patient with COVID-19 pneumonia (**left**) and after 6 months (**right**), with a substantial decrease noted (from 2.91 to 2.69 cm). Horizontal green lines represent the MPAD, whereas vertical red and green lines represent the distance of MPAD from the bifurcation of the pulmonary artery.

All patients intravenously received iodinised contrast agent following the CT pulmonary angiography (CTPA) protocol. The CT images were interpreted by two specialised radiologists who had extensive experience in chest imaging and were blinded to the clinical background of the patients. The metrics of the PA were extracted and the lung parenchymal findings were described, both in the acute phase of the infection and after the remission of the disease. The main PA diameter (MPAD) was measured at the level of the PA bifurcation at the level of the mid-aortic arch and the main carina (Figure 2); it was then classified according to the four-tier severity classification system of PA metrics on CT for the diagnosis and prognosis of PH, as previously described: normal if <2.7 cm for females or <2.9 cm for males, mild if ≥2.7 cm and <3.1 cm for females or ≥2.9 cm and <3.1 cm for males, moderate if ≥3.1 cm and <3.4 cm for both genders, and severe if ≥3.4 cm for both genders [18].

Statistical analysis was conducted using the software GraphPad Prism 9^®^. The non-parametric Wilcoxon matched-pairs signed rank test and the non-parametric Kruskal–Wallis test were used, while correlations were performed with the non-parametric Spearman correlation test. In all tests, a *p*-value of less than 0.05 was considered to be statistically significant.

## 3. Results

**Figure 3:**
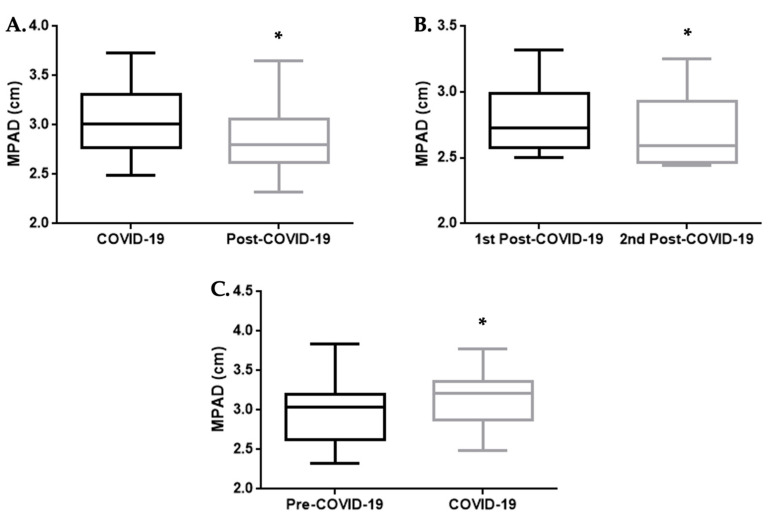
Boxplots of the comparisons of the value of the MPAD between different time points. (**A**) Comparison of the MPAD (cm) on chest CT of patients during the acute phase of COVID-19 (**left**) and in the post-COVID-19 state (**right**). The Wilcoxon test showed a statistically significant difference, with a *p*-value of <0.0001. (**B**) Comparison of the MPAD (cm) in consecutive chest CT scans of patients in the post-COVID-19 state. The Wilcoxon test showed a statistically significant difference, with a *p*-value of 0.0313. (**C**) Comparison of the MPAD (cm) on the chest CT of patients in the pre-COVID-19 state (**left**) and during the acute phase of COVID-19 (**right**). The Wilcoxon test showed a statistically significant difference, with a *p*-value of 0.0371. In each boxplot, the middle line represents the median value of the MPAD, while the upper and lower lines represent the maximum and minimum values, respectively, and the middle box represents the interquartile range. * *p*-value: <0.05.

The study population consisted of 48 adult individuals, 62.5% males and 37.5% females. In 31 patients, the mean ± standard deviation (SD) of the MPAD was 3.1 ± 0.3 cm in the acute phase of COVID-19 and 2.8 ± 0.3 cm in the post-infectious state after 2–18 months, as revealed on serial chest imaging. The difference in the MPAD between the two timepoints was statistically significant, with a *p*-value of <0.0001 (Wilcoxon test, Figure 3A). Moreover, in six patients in whom more than one post-COVID-19 chest CT scan was performed with a difference of 1–14 months, a further decline in the diameter with a *p*-value of 0.0313 was observed (Wilcoxon test, Figure 3B). In this subgroup of patients, the mean ± SD in the MPAD in the first post-COVID-19 chest CT was 2.8 ± 0.3 cm, and it was 2.7 ± 0.3 cm in the second.

A chest CT was also performed before SARS-CoV-2 infection, under other medical indications, in 10 patients from the total pool of 48 patients. We separately compared the findings in the acute phase of the disease with those observed in the pre-COVID-19 chest imaging for those cases. The difference between the MPAD measurements in the two timepoints was again statistically significant with a *p*-value of 0.0371 (Wilcoxon test, Figure 3C). In this subgroup of patients, the mean ± SD of the MPAD in the pre-COVID-19 chest CT was 3.0 ± 0.4 cm and it was 3.2 ± 0.4 cm in the COVID-19 CT.

**Figure 4:**
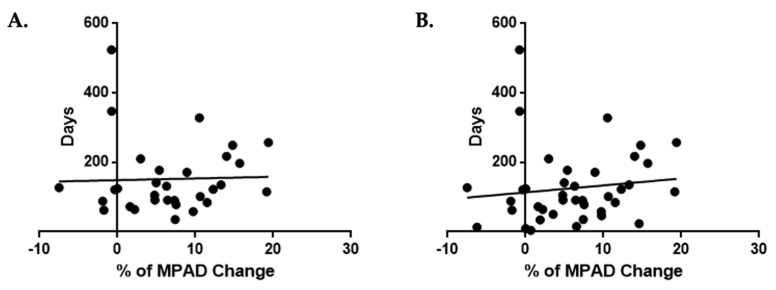
Diagrammatic representation of the relation between the time and the value of MPAD change. (**A**) Correlation of the percentage of change in the MPAD (%) between the chest CT scans performed during the acute phase of COVID-19 and in the post-COVID-19 state (2 to 18 months later) and the time that had elapsed from the acute phase, in a total of 31 patients. The Spearman test did not show a statistically significant correlation with r: 0.1791 and *p*-value: 0.3352. (**B**) Correlation of the percentage of change in the MPAD (%) between the chest CT scans performed during the acute phase of COVID-19 and in the post-COVID-19 state (less than a month to 18 months later) and the time that had elapsed from the acute phase, in a total of 39 patients. The Spearman test did not show a statistically significant correlation with r: 0.2270 and *p*-value: 0.1645.

In the 31 patients, we further assessed whether the percentage of change in the MPAD was associated with the time that had elapsed from the COVID-19 to the post-COVID-19 chest imaging, and we found no statistically significant correlations (Spearman test, r: 0.1791, *p*-value: 0.3352, Figure 4A). Even when we included in our analysis 8 additional cases from the total pool of 48 patients with a post-COVID-19 chest CT that was performed less than 2 months after the initial chest imaging, we did not observe any statistically significant correlations (Spearman test, r: 0.2270, *p*-value: 0.1645, Figure 4B).

**Figure 5:**
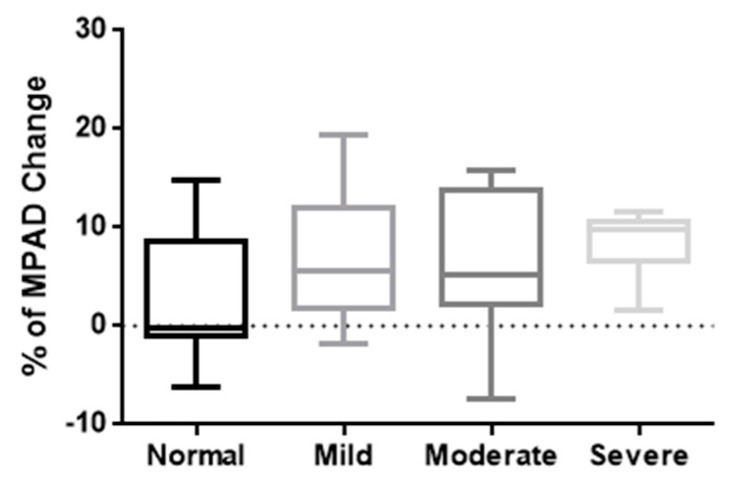
Boxplots of comparison of the values of MPAD change between different groups of PA enlargement severity. Comparison of the percentage of change in the MPAD (%) between the chest CT scans performed during the acute phase of COVID-19 and in the post-COVID-19 state, among the four groups of PA enlargement severity, as revealed in the first chest CT. Note the uptrend in the median percentage of change in the MPAD and the downtrend in the interquartile range from milder to more severe enlargement groups. The Kruskal–Wallis test, however, did not show a statistically significant difference, with a *p*-value of 0.2658. In each boxplot, the middle line represents the median value of the MPAD, while the upper and lower lines represent the maximum and minimum values, respectively, and the middle box represents the interquartile range.

Finally, the 39 cases in which a chest CT was available both during and after COVID-19 were categorised into four groups according to the degree of PA enlargement that was revealed on the first chest imaging examination, as described in the Materials and Methods section. The percentage of change in the MPAD (mean ± SD) was 3.1 ± 6.8 in the group with normal dimensions (10 individuals), 7.1 ± 7.3 in the group with mild enlargement (10 individuals), 6.6 ± 7.0 in the group with moderate enlargement (12 individuals), and 8.3 ± 3.5 in the group with severe enlargement (7 individuals). Interestingly, from the milder to more severe enlargement of the PA during the acute phase of the disease, there seemed to be an increase in the median percentage of change in the MPAD on repeat chest imaging, with an opposite trend in the interquartile range. However, when we compared the four groups with the Kruskal–Wallis test, no statistically significant differences were seen (*p*-value: 0.2658) (Figure 5).

## 4. Discussion

Medical imaging is of high importance to investigate COVID-19 and its deleterious post-infectious effects. Many studies concerning the CT abnormalities in acute COVID-19 and long COVID have been conducted. Most of them have focused on the parenchymal lesions that occur during the acute phase of the infection or afterwards, such as consolidations and GGOs in COVID-19 and associated secondary infections [19] or pulmonary fibrosis following COVID-19 pneumonia [20]. Of note, Colombi et al. described a correlation between the remission of the parenchymal lesions on follow-up chest CT scans and the pulmonary function testing (PFT) of the patients. More specifically, a larger percentage of low-attenuation areas on the chest CT was inversely correlated with the parameters of the PFT of the patients [21]. The observational study of Cavallari Strozze Catharin et al. included radiological and pulmonary function data and the timing of their normalisation after COVID-19. Half of the individuals included presented the normalisation of their chest CT in a time of less than 6 months, while in more than half of them, the PFT returned to normal in the same time period [22].

The enlargement of the PA is a well-described structural finding of chest CT in PH that is caused by a wide range of other non-COVID-19 clinical conditions; it has also been previously shown to be associated with both the severity and the duration of the disease. In 2020, Spagnolo et al. were the first to describe the presence of an enlarged PA in COVID-19 patients, showing a higher-than-normal PA maximal diameter and PA-to-aorta ratio; this was measured using chest CT in a small cohort of 44 COVID-19 patients in Italy. Remarkably, they included in their study only patients that had a previous (pre-COVID-19) chest CT and pointed out that the values measured were significantly increased in subsequent chest imaging performed during active disease [17].

Esposito et al. then observed similar findings in a large cohort of 1461 COVID-19 patients in Northern Italy, with an increased MPAD of >31 mm in the patients within 72 h of admission; they further revealed its association with a higher in-hospital mortality rate [23]. Erdogan et al. and Zhu et al. also reported increased mortality in 255 hospitalised Turkish and 180 hospitalised Chinese COVID-19 patients, respectively, that presented with an enlargement of the PA [24,25,26]. Furthermore, Yildiz et al. found that an increased PA diameter was correlated with the severity of COVID-19, as defined by clinical and laboratory findings, e.g., arterial oxygen saturation, body temperature, and inflammatory markers, in 101 Turkish patients [27]. In another study by Li et al. that compared 106 patients with COVID-19 and 52 patients with influenza virus pneumonia in China, an enlargement of the PA was present only in COVID-19 patients; thus, this pattern could differentiate the two entities and guide diagnosis by means of medical imaging [28]. In their study, concerning the findings in CTPA, i.e., pulmonary embolism and parenchymal abnormalities, Jalde et al. also noted that a larger MPAD positively correlated with extended parenchymal abnormalities, high C-reactive protein levels, and a poor disease outcome in 130 patients in Sweden [29]. Finally, the MPAD of hospitalised COVID-19 patients on admission significantly correlated with the post-COVID-19 functional scale (PCFS) status at two-month follow-up, in addition to other prognostic parameters, as shown in a study by Ismail et al. including 465 patients in Lebanon [30].

In agreement with the scientific evidence provided by previous related studies, in this cohort, the enlargement of the PA was a common imaging finding in COVID-19, as revealed on chest CT. This was evident from both the comparison of the MPAD during the acute phase of the infection with reference values and its comparison with the baseline measurements in cases in which a pre-COVID-19 chest CT was available. What had not been previously investigated, namely the progression of PA enlargement in COVID-19 and afterwards, was at the epicentre of this study. Interestingly, it was clearly shown that the increase in the MPAD regressed after the remission of the disease, suggesting that this particular structural alteration is only acutely observed during the infection. A further decline in the MPAD was even noted in cases in which a second post-COVID-19 chest CT was performed. A clear association of the degree of PA enlargement with disease severity and the mortality rate was previously described in several studies, as mentioned above. We further showed that the severity of PA enlargement tends to determine the degree of its regression in the post-infectious state. More severe enlargements of the PA were associated with greater percentages of regression, despite having a lack of statistical significance; this is probably due to the relatively small sample size, especially in the group with the severe enlargement of the PA. Our findings indicate that the enlargement of the PA is an acute and reversible cardiovascular event in COVID-19, in the context of extensive pulmonary microvascular defects and the consecutive dilatation and strain of the right cardiac chambers, which are attributed both to direct viral insult and a systemic inflammatory response. Since all patients were free of clinical manifestations at the time of the follow-up chest CT in which PA enlargement was found to be reversed, we suggest that this regression could constitute a potential marker for the remission of the disease.

The limitations of this study included the relatively small sample size, the heterogeneity of the time that had elapsed between the infection and the performance of the chest CT scans during the acute phase and the post-infectious state among different participants, the relatively small number of patients in the group with a severe enlargement of the PA, and the lack of correlations between the clinical and laboratory features of the disease during testing. The participants in our study population consisted of a diverse group regarding their need for hospitalisation, hospitalisation in a ward or intensive care unit, the need for supplementary oxygen therapy, and the medications they received, i.e., anticoagulants, antivirals, corticosteroids, etc. The thorough collection and correlation of these clinical data with the change in the MPAD and the time needed for the enlargement to regress would provide more information about the behaviour of this potential marker for the remission of the disease. Moreover, during this study, no investigation of any potential correlations between the specific strain of the virus or the progression of the parenchymal lesions with the value of the MPAD was performed. Should these aforementioned factors of the study become readjusted, more robust results could contribute to a greater understanding of the aspects of pulmonary vascular involvement and complications in COVID-19.

## 5. Conclusions

A few studies in the currently available literature concern the pulmonary vascular abnormalities revealed on chest CT during the acute phase of SARS-CoV-2 infection or the post-COVID-19 state. However, an agreement is drawn in all these studies regarding the enlargement of the MPAD in COVID-19, while no data are available to date regarding the evolution of the phenomenon after the remission of the disease.

In our study group, in concordance with the literature, the enlargement of the PA during the acute phase of COVID-19 was observed. Interestingly, we further assessed this vascular change in the post-COVID-19 state in individuals who were free of symptoms and showed that it regresses. The insignificant correlations between the regression and time and the severity of the enlargement could be attributed to the small size of our sample.

In conclusion, the enlargement of the PA is a common finding in COVID-19 pneumonia, which is associated with the microangiopathy derived from SARS-CoV-2 infection, accompanies the acute phase of the disease and regresses after its remission. Further studies are needed to shed more light on the course of this apparently reversible cardiovascular event, as well as to investigate its clinical significance in the remission of the disease or its progression to long COVID.

## Data Availability

The data presented in this study are available upon request from the corresponding author.
